# Supplementary material for: ProteinShader: illustrative rendering of macromolecules
Source: BMC Struct Biol. 2009 Mar 30;9:19. doi: 10.1186/1472-6807-9-19 (PMC2672931; doi:10.1186/1472-6807-9-19)
Supplement: Additional file 1 — ProteinShader program without source code. This compressed file contains the complete ProteinShader program including associated libraries, but no source code. A README.txt file gives an overview of the ProteinShader distribution, and the index.html file in the help subdirectory has directions on getting started with the program as well as a set of tutorials. [file 1472-6807-9-19-S1.zip › ProteinShader-beta-0_9_4-binary/help/api/org/proteinshader/gui/listeners/menubar/PositionMenuListenerFactory.html]

PositionMenuListenerFactory (ProteinShader API)


|  |  |  |
| --- | --- | --- |
| **Overview** \| **Package** \| **Class** \| **Use** \| **Tree** \| **Deprecated** \| **Index** \| **Help** | |  |
| **PREV CLASS**   **NEXT CLASS** | **FRAMES**    **NO FRAMES**     **All Classes** |  |
| SUMMARY: NESTED \| FIELD \| CONSTR \| METHOD | DETAIL: FIELD \| CONSTR \| METHOD |  |


---


## org.proteinshader.gui.listeners.menubar Class PositionMenuListenerFactory

```
java.lang.Object
  org.proteinshader.gui.listeners.menubar.PositionMenuListenerFactory
```

---

``` public class PositionMenuListenerFactory extends Object ```

Creates all event listeners needed by the PositionMenu of the
MainMenuBar.

---

**Constructor Summary**

`PositionMenuListenerFactory(Mediator mediator)`
  
          Constructs a PositionMenuListenerFactory.


| **Method Summary** | |
| --- | --- |
| `ActionListener` | `createPositionMenuItemsListener()`             Creates an action listener for the PositionEnum menu items. |

**Methods inherited from class java.lang.Object**

`clone, equals, finalize, getClass, hashCode, notify, notifyAll, toString, wait, wait, wait`

**Constructor Detail**

### PositionMenuListenerFactory

```
public PositionMenuListenerFactory(Mediator mediator)
```

:   Constructs a PositionMenuListenerFactory.

    **Parameters:**: `mediator` - the centralized Mediator object that listeners call on to accomplish their task.


**Method Detail**

### createPositionMenuItemsListener

```
public ActionListener createPositionMenuItemsListener()
```

:   Creates an action listener for the PositionEnum menu items. When
    the action listener is called, it call on the setImagePosition of
    the Mediator.

    :   **Returns:**: The action listener.


---


|  |  |  |
| --- | --- | --- |
| **Overview** \| **Package** \| **Class** \| **Use** \| **Tree** \| **Deprecated** \| **Index** \| **Help** | |  |
| **PREV CLASS**   **NEXT CLASS** | **FRAMES**    **NO FRAMES**     **All Classes** |  |
| SUMMARY: NESTED \| FIELD \| CONSTR \| METHOD | DETAIL: FIELD \| CONSTR \| METHOD |  |


---

# *Copyright © 2007-2008*
